# CGRP and CGRP receptors in human and rhesus monkey cerebellum

**DOI:** 10.1186/1129-2377-14-S1-P91

**Published:** 2013-02-21

**Authors:** S Eftekhari, CA Salvatore, BM Connolly, S O’Malley, PJ Miller, Z Zeng, L Edvinsson

**Affiliations:** 1 Department of Clinical Sciences, Division of Experimental Vascular Research, Lund University, Lund, Sweden; 2 Department of Pain and Migraine Research,West Point, PA, USA, USA; 3 Department of Imaging, Merck Research Laboratories, West Point, PA, USA, USA; 4 Department of Clinical Sciences, Division of Experimental Vascular Research, Lund University, Lund, Sweden

## Background

The cerebellum is classically considered mainly involved in motor processing, but recent studies have suggested several other functions, including pain processing. PET studies of acute migraine attacks have revealed activation of the cerebellum. In human pain imaging studies activation of the cerebellum is almost always observed, suggesting a role in nociception. Calcitonin gene-related peptide (CGRP) has been shown to be one of the most important neuropeptides involved in migraine pathology, where there is elevated release of CGRP during migraine attacks and CGRP receptor antagonists have antimigraine efficacy.

## Methods

In vitro autoradiography mapping studies were performed on human and rhesus monkey. Slices of cerebellum were incubated with [3H]MK-3207 (a CGRP receptor antagonist) or [125I]CGRP to define the binding sites. Immunofluorescence was used to study the detailed distribution of CGRP and its receptor components- calcitonin receptor-like receptor (CLR) and receptor activity modifying protein 1 (RAMP1)- in human and rhesus monkey cerebellum, using a set of newly characterized antibodies. In addition, expression of procalcitonin was studied.

## Results

High [3H]MK-3207 binding densities were observed in the molecular layer of rhesus cerebellum, however due to the limit of resolution of the autoradiographic image the exact cellular localization could not be determined. Similarly, [125I]CGRP binding was observed in the molecular layer of human cerebellum. Immunofluorescence revealed expression of CGRP, CLR and RAMP1 in the Purkinje cells and cells in the molecular layer. Procalcitonin was also found in Purkinje cells and cells in the molecular layer.

## Conclusions

The study demonstrated CGRP receptor binding sites and expression of CGRP and its receptor in primate cerebellum, which points toward a functional role of CGRP in cerebellum. It is also suggests that cerebellum may be a site of action of CGRP receptor antagonists.

